# Heterogeneously Catalyzed Continuous-Flow Hydrogenation Using Segmented Flow in Capillary Columns

**DOI:** 10.1002/cctc.201100044

**Published:** 2011-06-09

**Authors:** Jasper J W Bakker, Martijn M P Zieverink, Raf W E G Reintjens, Freek Kapteijn, Jacob A Moulijn, Michiel T Kreutzer

**Affiliations:** [a] Department of Chemical Engineering, Delft University of TechnologyJulianalaan 136, 2628 BL (The Netherlands), Fax: (+31) 015 278 5006 E-mail: m.t.kreutzer@tudelft.nl; [b] R.W.E.G. Reintjens, DSM Pharmaceutical Products ASC&DP.O. Box 18, 6160 MD Geleen (The Netherlands)

**Keywords:** azides, flow reactions, heterogeneous catalysis, palladium, pharmaceutical intermediates

This paper explores how the visible features of segmented-flow, under reaction conditions, can be used in lab-scale multiphase heterogeneous catalysis. Continuous-flow microreactors are now routinely used in bench-scale synthesis[1] and optimization[2] applications, owing to their small reactant inventory, negligible heat effects at small scales, and fast mixing.[3] However, miniaturizing multiphase heterogeneous catalysis on chips is considerably more difficult than homogeneous liquid phase chemistry. Several applications of gas–liquid[2b, 4] and gas–liquid–solid[5] reactions have been reported. In these continuous-flow devices, the catalyst was immobilized on the wall of the channel[5a, 6] or incorporated as powder.[5e, 7] A powder packed-bed gas–liquid microreactor may appear ideal for off-the-shelf catalysts, but in practice such reactors are cumbersome: critical packing parameters vary from one instance to the next and channeling and flow hysteresis abound, as we have recently visualized.[8] For immobilized catalysts, Kobayashi et al. have advocated creating a thin film of liquid on the walls, sheared along by a fast-flowing gas stream.[5a] A drawback of this system is that it is hard to control or visualize how long the reactants are in contact with the catalyst, because both phases each move at their own velocity. Especially for more complex pathways, the spread in residence time reduces yields.

**Figure 1:**
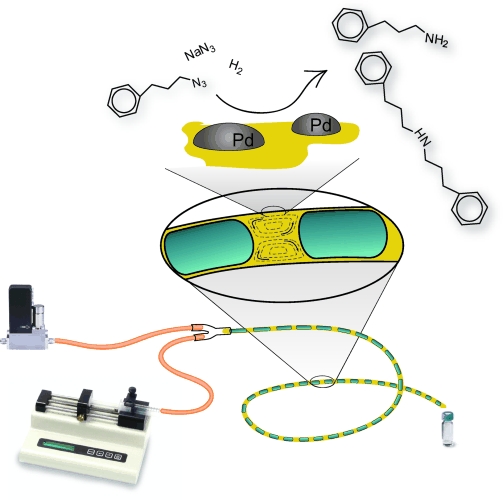
Continuous capillary microreactor with a Pd catalyst immobilized on the inner wall operated in segmented gas–liquid flow.

Here, we overcome monitoring and yield problems by using wall-catalyzed segmented flow (Figure 1). Flow segmentation has found widespread use in liquid–liquid[4c, 9] and gas–liquid[4d, 10] applications, motivated by good contact between fluid phases. In this work, we highlight that the flow pattern also enhances contact with the catalyst on the wall, inspired by our work in pilot-plant studies for monolith-based reactors.[11] We focus on using standard capillary columns, readily available to the bench chemist. The uncomplicated construction and, equally important, simple visual monitoring will be a powerful tool in the hands of synthetic chemists.

**Figure 2:**
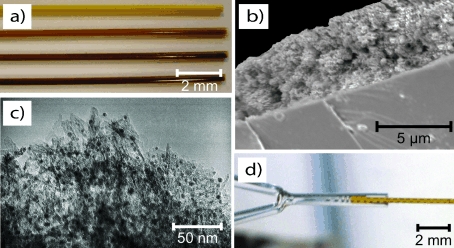
a) Darkening of a capillary after Pd impregnation (0, 1.1, 2.7, 5.7 wt % Pd); b) SEM micrograph of porous γ-Al_2_O_3_ layer coated on the inner wall of the capillary; c) TEM micrograph of 5 nm Pd particles (black dots) supported on γ-Al_2_O_3_ (5.7 wt % Pd); d) Formation of segmented flow at the capillary inlet.

We used commercially available fused-silica capillaries coated with a 6 μm thick layer of high surface area γ-Al_2_O_3_ that we pretreated and impregnated with a [Pd(OAc)_2_] solution (Figure 2 and see the Supporting Information for details). This resulted in nanosized Pd particles evenly dispersed on the γ-Al_2_O_3_ coating layer (Figure 2 c). We tested their activity with the well-studied hydrogenation of cyclohexene.[12] In a 17 cm capillary (residence time ≈4 s) the conversion was 43 % at 20 °C, without deactivation, increasing to >99 % for a 50 cm piece.

**Figure 3:**
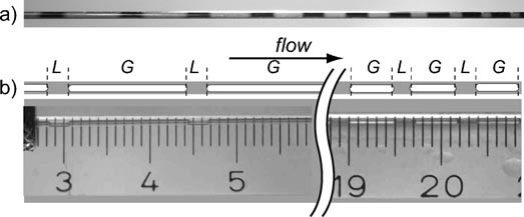
a) Visual observation of shrinking bubbles during a hydrogenation reaction in a Pd-capillary; b) Measurement of the bubble length from photographs at the inlet and outlet of a capillary.

The segmented flow regime (Figure 2 d) is an especially useful flow pattern in synthesis because the chemistry can be followed visually.[4b, 9c] From the velocity of the bubbles, the reaction time is determined, and the conversion rate is seen from the decreasing length of the bubbles, as they move through the capillary (Figure 3). We fully exploit this fact by visually measuring the conversion accurately, provided that conditions are chosen such that hydrogen consumption is significant. Bubble-to-bubble variation requires that several tens of bubbles are measured, but then, the visual method and GC-analysis agree within 5 % (details in the Supporting Information). Rapidly available quantitative kinetic data thus allows for fast fine-tuning to maximize yields or minimize deactivation.

Before we discuss such an optimization, we first show that segmented flow ensures an optimal yield. Previously, we reported that mass transfer to the wall in segmented flow is extremely fast.[11] We confirm this by measuring the activation energy of the hydrogenation of cyclohexene (details in the Supporting Information). In our capillary, we determined that the activation energy was 34 kJ mol^−1^ at *T* <330 K, which is in agreement with literature[13] and rules out transport limitations inside or outside the porous alumina layer, which would have lowered this value.

**Scheme 1:**
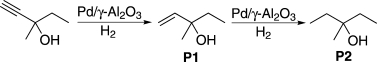
Selective hydrogenation of 3-methyl-1-pentyn-3-ol over a Pd catalyst to the desired 3-methyl-1-penten-3-ol (**P1**) and the overhydrogenation to 3-methyl-3-pentanol (**P2**).

**Figure 4:**
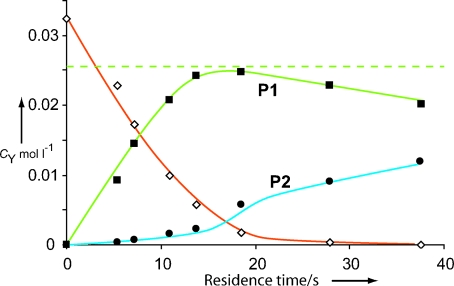
Effluent concentrations as a function of residence time of the hydrogenation of 3-methyl-1-pentyn-3-ol in ethanol over a 0.02 wt % Pd catalyst operated in segmented-flow at 24 °C. The dashed line shows the maximum obtainable yield.

Apart from eliminating diffusional effects, segmented flow hardly exhibits axial dispersion,[14] which is notorious for low yields in continuous-flow reactors and polydisperse particles in continuous crystallization.[15] We illustrate the improvement with the selective Pd-catalyzed hydrogenation of 3-methyl-1-pentyn-3-ol to **P1** (Scheme 1) without over-hydrogenation to **P2**. Kinetic modeling showed that, for a feed of 0.032 mol L^−1^, the maximum possible yield is 78–81 %, which is obtained only at precisely the right residence time.[16] Any distribution of residence time reduces the yield. In a one-day optimization, we approach the theoretical optimum, obtaining a yield of **P1** (78±2) % using segmented flow (Figure 4). Without H_2_ bubbles, the yield of **P1** would have been about 57 %.

**Scheme 2:**
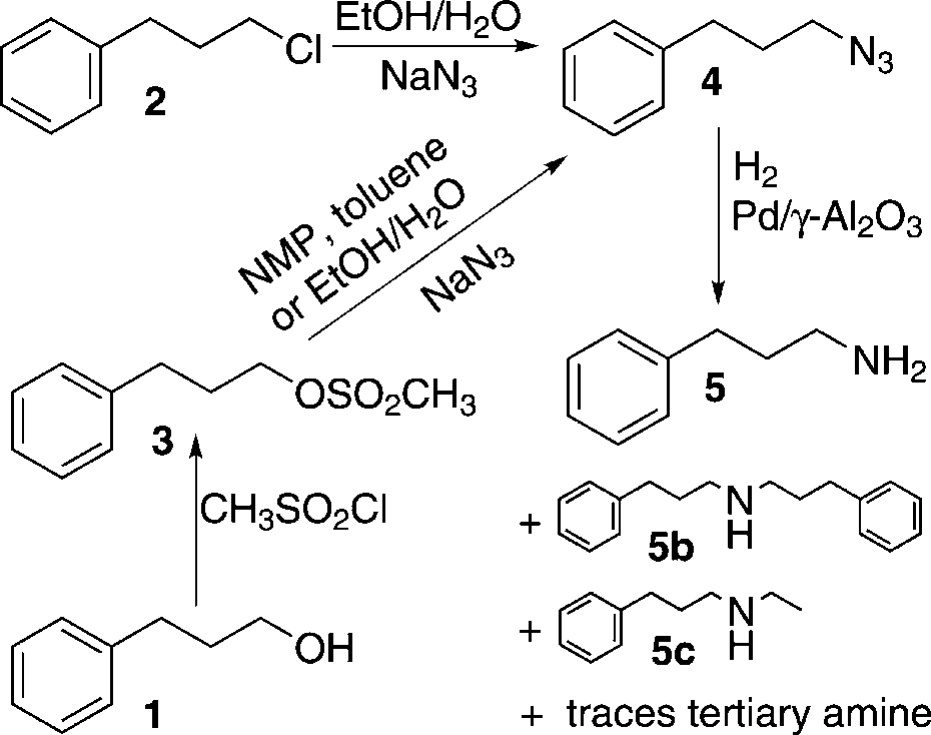
Synthesis routes for 3-phenyl-propyl-amine **5**.

Now that we have established the benefits of segmented flow in Pd-capillaries for single step hydrogenation reactions, we return to rapid optimization of a multistep synthesis. As an example to demonstrate the benefit of segmented flow, we synthesized primary amine **5** by hydrogenation of the intermediate azide **4** (see Scheme 2). The hydrogenation of the nitrogen-containing compounds over Pd catalysts is a good example of strongly adsorbing, or even poisoning, reactants and products. Such deactivation is better analyzed in continuous flow than in repeated batch experiments. Moreover, azide chemistry benefits greatly from miniaturized synthesis, because the toxic and explosive properties of azides complicate the handling of large quantities. Azide **4** was synthesized from **3** or **2** in various solvents, and subsequently hydrogenated without intermediate workup, i.e., purification was postponed until the toxic and explosive **4** was converted (details in the Supporting Information). The optimization problem, then, is to keep the hydrogenation to the primary amine **5** going as long as possible until deactivation of the catalyst by strongly adsorbing compounds in the synthesis mixture.

**Figure 5:**
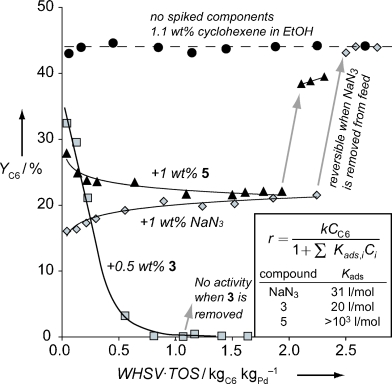
Spiking of cyclohexene hydrogenation at 24 °C with components from Scheme 2 to determine the strength and reversibility of adsorption on the Pd catalyst.

We investigated how much of the various compounds deactivate Pd, one by one, by measuring how much they reduce the rate of the hydrogenation of cyclohexene—in the azide hydrogenation, bubbles do not shrink owing to the formation of N_2_, so we used a simple test reaction that does exhibit bubble shrinkage because it does not produce a gas. This works because we are predominantly interested in the adsorption strength for the various deactivating compounds, which enters in the same way into the rate expression for the hydrogenation of both azide and cyclohexene. These *spiking* experiments are cumbersome in batch, but easy in our continuous system. Now, only the conversion needed to be monitored to determine the adsorption strength *K* of the various components on the catalyst, and switching to an unspiked feed shows whether adsorption is reversible (Figure 5). Rapidly, the population balance on the Pd surface for these complex synthesis mixtures was determined and used in the optimization of the hydrogenation process and synthesis route.

Based on these rapid spiking experiments, we found that **3** adsorbs irreversibly, whereas azides, including residual NaN_3_, adsorb reversibly. Adsorption of **5** was minimized by H_2_O in the feed, because the ammonium complex that formed hardly adsorbs. Condensation reactions, giving **5 b** and **5 c**, occurred less at high dilution and low temperature, and were solvent-sensitive. Toluene as solvent competed significantly with **4** for adsorption on the Pd surface, whereas ethanol enhanced condensation. An optimum of 2 kg of amine **5** could be synthesized using 1 g Pd, using **2** as starting material, in ethanol/H_2_O without catalyst regeneration (entry 7, Table S2 in the Supporting Information). This was achieved in the same capillaries, showing that kilolab quantities can readily be made in the same bench-scale equipment, with only longer time-on-stream.

Segmented-flow accelerates optimization of continuous-flow hydrogenations using heterogeneous catalysis. Conversion can be monitored visually, which gives hands-on control over the activity and deactivation of the catalyst. Reagents exchange rapidly with the catalyst and without axial dispersion, yields are the same as in batch. We have shown that continuous-flow analysis allows a fast optimization of various aspects of heterogeneous catalysis and synthesis routes, such as solvent effects, competitive adsorption and irreversible poisoning.
