# Supplementary material for: Heterogeneously Catalyzed Continuous-Flow Hydrogenation Using Segmented Flow in Capillary Columns
Source: ChemCatChem. 2011 Jun 9;3(7):1155–7. doi: 10.1002/cctc.201100044 (PMC3149126; doi:10.1002/cctc.201100044)
Supplement: Supplementary file 1 [file cctc0003-1155-SD1.pdf]

Heterogeneous & Homogeneous & Bio-
CHEMCATCHEM
CATALYSIS

Supporting Information

© Copyright Wiley-VCH Verlag GmbH & Co. KGaA, 69451 Weinheim, 2011

Heterogeneously Catalyzed Continuous-Flow Hydrogenation Using Segmented Flow in Capillary Columns

Jasper J. W. Bakker,[a] Martijn M. P. Zieverink,[a] Raf W. E. G. Reintjens,[b] Freek Kapteijn,[a] Jacob A. Moulijn,[a] and Michiel T. Kreutzer*[a]

cctc_201100044_sm_miscellaneous_information.pdf

Table of Contents

1. Capillary preparation and characterization…………………………………………………………...1

2. Hydrogenation experiments……………………………………………………………………..3

3. 3-methyl-1-pentyn-3-ol hydrogenation…………………………………………………….…5

4. Cyclohexene hydrogenation……………………………………………………………………..8

5. 3-phenyl-propyl-azide hydrogenation…………………………………………………………...9

1. Capillary preparation and characterization

A commercially available fused silica capillary column with an inner diameter of 530 μm coated with a 6 μm γ-Al_2O_3 (supplied by Restek: Rt-Alumina™ PLOT column, aluminum oxide Porous Layer Open Tubular column) resulting in an open inner diameter of 518 μm were used as a microreactor. The thickness of the fused silica capillary wall was 50 μm, and the outer coating of polyimide was 20 μm thick. This polyimide coating provides increased elasticity, flexibility, and tensile strength to the fused silica column and it can withstand temperatures up to 350°C in air or 400°C in N_2.

Prior to catalyst preparation, the capillaries were heated for 4 h at 260°C with a heating rate of 2 K min^{-1} to re-activate the deactivated γ-Al_2O_3 (these columns were partly deactivated with Na_2SO_4 by the supplier), rinsed with water (Milli-Q) at 1 ml min^{-1} for 30 min followed by a flow of 1 ml(STP) min^{-1} N_2 for 30 min and subsequently dried under flowing air in an oven for 2 h at 120°C.

A solution of Palladium (II) acetate ($Pd(OAc)_2$, Alfa Aesar, 45.9 wt% Pd) in toluene (J.T. Baker) was prepared and stirred for 60 min. The capillaries were filled with the $Pd(OAc)_2$ solution, capped on both sides with GC septa and ion-exchange occurred for 24 h. Capillaries were impregnated with solutions of different $Pd(OAc)_2$ amounts aiming for Pd catalysts between 0.003 and 7 wt% Pd on γ-Al_2O_3.

In a typical experiment, the amount of Pd in the solution was aimed at 7 wt% per unit γ-Al_2O_3 weight. After the impregnation the capillary was emptied and washed with *n*-hexane followed by flowing N_2 for 20 min at room temperature. After flushing, they were dried and calcined with a flow of air for 1 h at 120°C, followed by 2 h at 250°C, and then cooled to room temperature, with a heating rate up and down of 2 K min^{-1}. Reduction of the Pd nanoparticles took place at room temperature for 12 h prior to a hydrogenation experiment. The H_2 flow and pressure during reduction was 1 ml(STP) min^{-1} and 1.1 bar.

The amount of Pd in a capillary was determined by atomic adsorption spectroscopy (AAS) on a Perkin-Elmer 4100ZL. Transmission electron microscopy (TEM) was performed using a Philips CM30T electron microscope with a LaB6 filament as a source of the electrons equipped with an energy dispersive X-ray Spectrometer. Scanning electron microscopy (SEM) was performed using a Philips XL20 electron microscope to investigate the thickness and homogeneity of the γ-Al_2O_3 layer. The γ-Al_2O_3 layer thickness was determined at several points in the capillary and was 6±0.5 μm. N_2 physisorption, performed on a Quantachrome Autosorb-6B, was used to determine the specific surface area S_{BET}, the average pore diameter, and the pore volume of the γ-Al_2O_3 coating. The S_{BET} of the γ-Al_2O_3 layer was 195 m^2 g^{-1}, the average pore diameter was 10 nm, the pore volume was 0.47 cc g^{-1}, and the porosity was 0.63.

AAS revealed that 80% of the aimed amount of Pd was deposited in the capillary resulting in 5.7 wt% Pd loading and all Pd was deposited with aimed amounts of <3 wt%. Pd nanoparticles (d = 5 nm, determined with TEM and CO chemisorption) were homogenously distributed over the entire length of a capillary of 0.5 m. Capillaries with lower wt% Pd resulted in smaller Pd nanoparticles (determined with TEM): for 1.1 and 2.7 wt% Pd capillaries the particles size of Pd was 2.4 and 3.3 nm, respectively.

2. Hydrogenation experiments

The fused silica capillaries with Pd nanoparticles deposited on the γ-Al_2O_3 layer were connected to a gas mass flow controller for the H_2 supply (Bronkhorst HI-TEC model F-200CV-FAC-33-V with a maximum of 1 H_2 ml(STP) min^{-1}) and to a syringe pump (Harvard, PHD2000 programmable) containing a 50 ml syringe (SGE Europe Ltd.) filled with a liquid reactant/solvent mixture. Simple quartz Y-mixer (Restek) and stainless steel T- and Y-mixers (Valco and Swagelok) were used as gas-liquid distributors which were attached upstream of a capillary.

Continuous flow experiments were performed in the segmented flow regime. The analysis of the reaction mixture effluent was only considered after a stable flow pattern of segmented flow had established inside the transparent capillary. Samples were only collected and analysed by HPLC and/or GC analysis when a stable segmented flow was observed.

Initially, the capillaries were reduced for 12 h at room temperature in a H_2 flow of 1 ml(STP) min^{-1}. Experimental results obtained in the non-steady state induction period were discarded. No improvement was found in the reaction rate and stability in comparison with capillaries that were not reduced in advance, and the time-consuming procedure was abandoned and capillaries were used as prepared. Before every hydrogenation experiment, the H_2 flow was switched on first and after 10 min the liquid flow was switched on. This short procedure resulted in capillaries that were fully active at the start of experiments.

The capillaries were submerged in a temperature controlled stirred water bath (IKA RCT basic and IKA ETS-D4 Fuzzy). The progress of reactions was measured using off-line GC analysis (also HPLC for the hydrogenation of 3-phenyl-propyl-azide) and also by visual observation of the H_2 bubble shrinkage during the hydrogenation of 3-methyl-1-pentyn-3-ol and cyclohexene.

A capillary of 0.5 m was cut into several pieces of equal length and these pieces were tested separately for cyclohexene hydrogenation activity. This gave hydrogenation conversions within 3% confirming that the Pd was distributed homogenously in the axial direction, as was also shown by TEM analysis of the

separate pieces. No (detectible) activity was observed for capillaries without Pd when performing the hydrogenation of cyclohexene, 3-methyl-1-pentyn-3-ol and 3-phenyl-propyl-azide.

The Pd content on the capillary was measured with AAS before and after all hydrogenation reactions and no decrease of the Pd content was observed except for the hydrogenation of 3-phenyl-propyl-azide in NMP. Note that the other solvents used in the hydrogenation 3-phenyl-propyl-azide (ethanol/water and toluene) showed no Pd content decrease. After reaction, several product mixtures were analysed by inductively coupled plasma optical emission spectroscopy (ICP-OES) and no Pd was detected in these mixtures (at least no Pd dissolution above the detection limit). ICP-OES was carried out with a Perkin-Elmer Optima 5300. Solutions of the products of the hydrogenations in the appropriate solvent were used as matrix in the calibration samples. TEM analysis of the post-mortem catalyst at several points did not show a particle size decrease or an accumulation of Pd (readsorption of leached Pd) at the end part of the capillary for the hydrogenation of 3-methyl-1-pentyn-3-ol, cyclohexene and 3-phenyl-propyl-azide in ethanol/water and toluene. Continuous experiments with increasing time on stream for the hydrogenation of cyclohexene and 3-methyl-1-pentyn-3-ol showed no deactivation under kinetically controlled conditions and capillaries could be used for several weeks without deactivation. The hydrogenation of 3-phenyl-propyl-azide showed strong deactivation which was mainly caused by poisoning (reversible and irreversible adsorption) except for the hydrogenation of 3-phenyl-propyl-azide in NMP, where the reaction mixture had a yellowish colour and the capillary became completely yellow again pointing towards full Pd dissolution without readsorption of leached Pd downstream or at the end of the capillary. AAS analysis of the used capillaries in the hydrogenation of 3-phenyl-propyl-azide in NMP showed that most Pd leached into the reaction mixture (less than 0.2 wt% left from the 5.7 wt% Pd originally on the capillary) caused by complex formation with components in the highly polar reaction mixture. Moreover, N_2 physisorption revealed that the S_{BET} of the γ-Al_2O_3 support decreased significantly from 195 to 100 m^2 g^{-1} indicating γ-Al_2O_3 sintering and/or dissolution because of the high pH (~10) of the reaction mixture. In addition, regeneration attempts by H_2 and N_2 flow at elevated temperatures did not result in any activity increase, indicating Pd loss for the hydrogenation in NMP.

3. 3-methyl-1-pentyn-3-ol hydrogenation: determination of the conversion by visual inspection of shrinking H_2 bubbles

Samples of 3-methyl-1-pentyn-3-ol hydrogenation (Scheme 1, Communication) were analyzed using a Varian CP-3380 GC with a Chrompack CP-SIL 8 CB (PBX5) column of 60 m (internal diameter 0.25 mm; injector temperature = 220°C; ramp = 10 K min^{-1}; FID temperature = 330°C). Retention times were: 7.8 min (3-methyl-1-pentyn-3-ol), 8.1 min (3-methyl-1-penten-3-ol), 9.6 min (3-methyl-3-pentanol), and 4.0 min (ethanol). 3-methyl-1-pentyn-3-ol, 3-methyl-1-penten-3-ol, 3-methyl-3-pentanol (Fluka), and dry ethanol (Aldrich) were used as received. Figure 1a shows the decreasing H_2 bubbles in a capillary during the hydrogenation of 3-methyl-1-pentyn-3-ol. A ruler bar was mounted behind the capillary and series of 30 H_2 bubbles were measured at the inlet and at the outlet. The H_2 bubble lengths were captured at the inlet and at the outlet of the capillary with a camera (Olympus i-speed 2, object-lens Nikon 55 mm) at a frame rate of 150 fps. Figure 1b shows the H_2 bubble length as a function of the position in the capillary, showing the (exponential) decay of H_2. Normally, when operating a tubular reactor at high conversions (integral conditions, concentration gradient over the length of the reactor) the form/reaction order of the rate equation is guessed, inserted in the design equation, integrated and plotted in a graph of concentration versus time in an iterative way. However, when the decay of H_2 bubble lengths is visually monitored, one can experimentally determine the reaction order of the gas phase.

a)

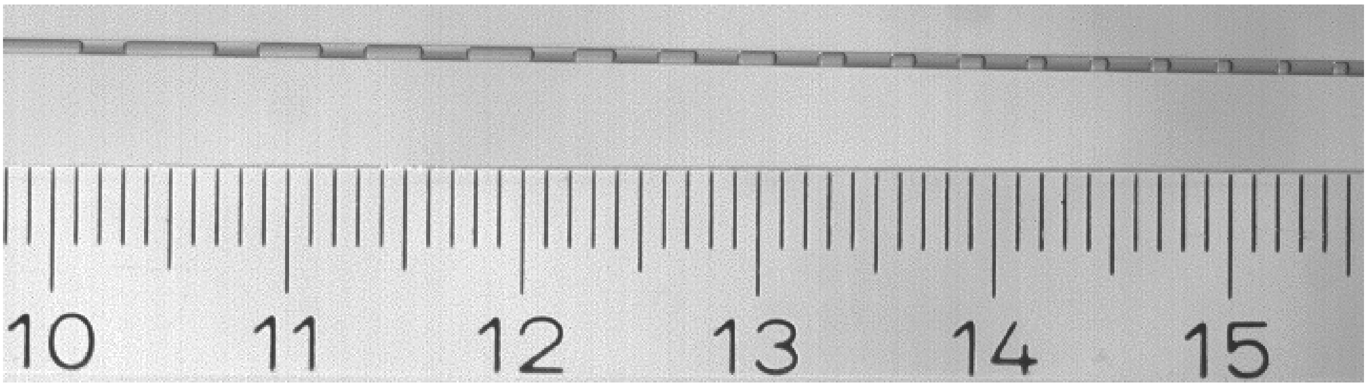

b)

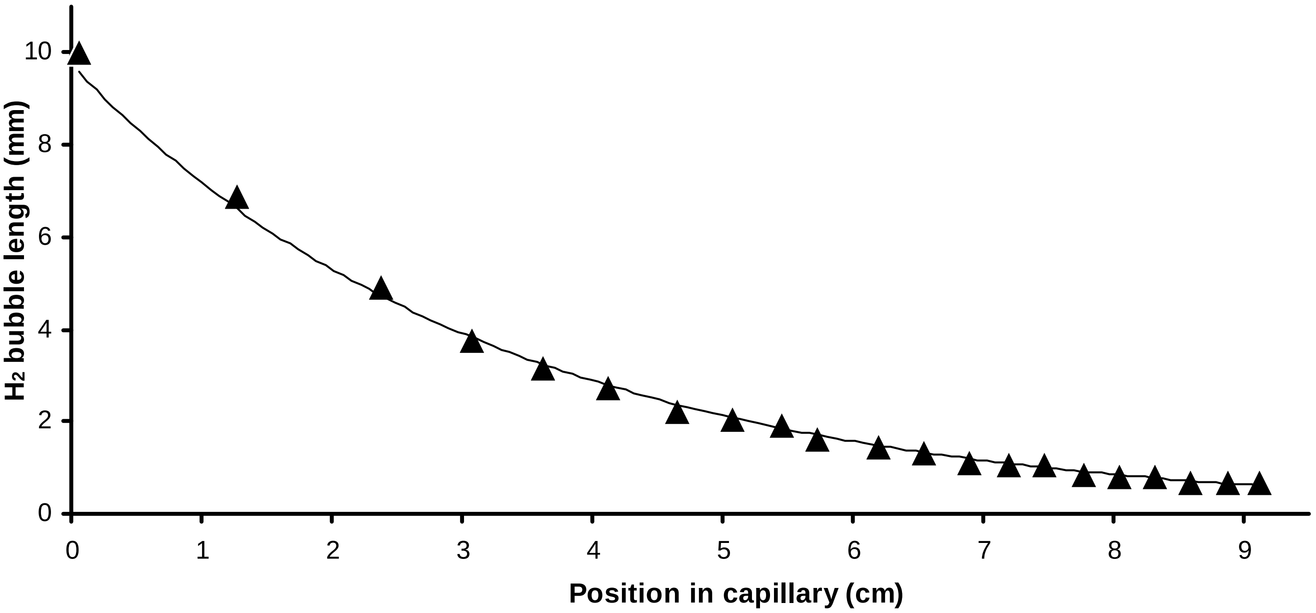

Figure 1. a) Visual observation of shrinking H_2 bubbles during the hydrogenation of 3-methyl-1-pentyn-3-ol in a Pd -capillary + ruler bar (cm). b) H_2 bubble length as a function of the position in the capillary. Conditions: 1.1 wt% Pd, T = 25°C; 1.1 bar H_2; $C_{Y,0}$ = 294 mol m^{-3}; u_L = 100 µl min^{-1}; and u_G = 470 µl min^{-1}.

Two examples are given where the conversion determined by GC analysis is compared with the conversion determined by visual inspection of shrinking H_2 bubbles. The conditions are given in Table 1. Figure 2 shows the H_2 bubble lengths that were obtained in these experiments. No H_2 bubble lengths were found outside the indicated range. In Figure 3 a comparison is shown of the conversion determined by GC analysis and the conversion determined from visually observed decreased H_2 bubble lengths after the

expansion of H_2 bubbles due to pressure drop in the capillary was accounted for. The pressure drop was calculated using a model developed by Kreutzer et al.[1]

Table 1. Conditions of examples of the calculation of conversion from H_2 bubble length.

	Run 1	Run 2
Temperature (°C)	26	26
3-methyl-1-pentyn-3-ol (mol m^{-3})	294	294
Capillary length (m)	0.25	0.25
Amount of Pd (wt%)	1.1	1.1
H_2 flow rate (µl(STP) min^{-1})	860	1000
Liquid flow rate (µl min^{-1})	100	200

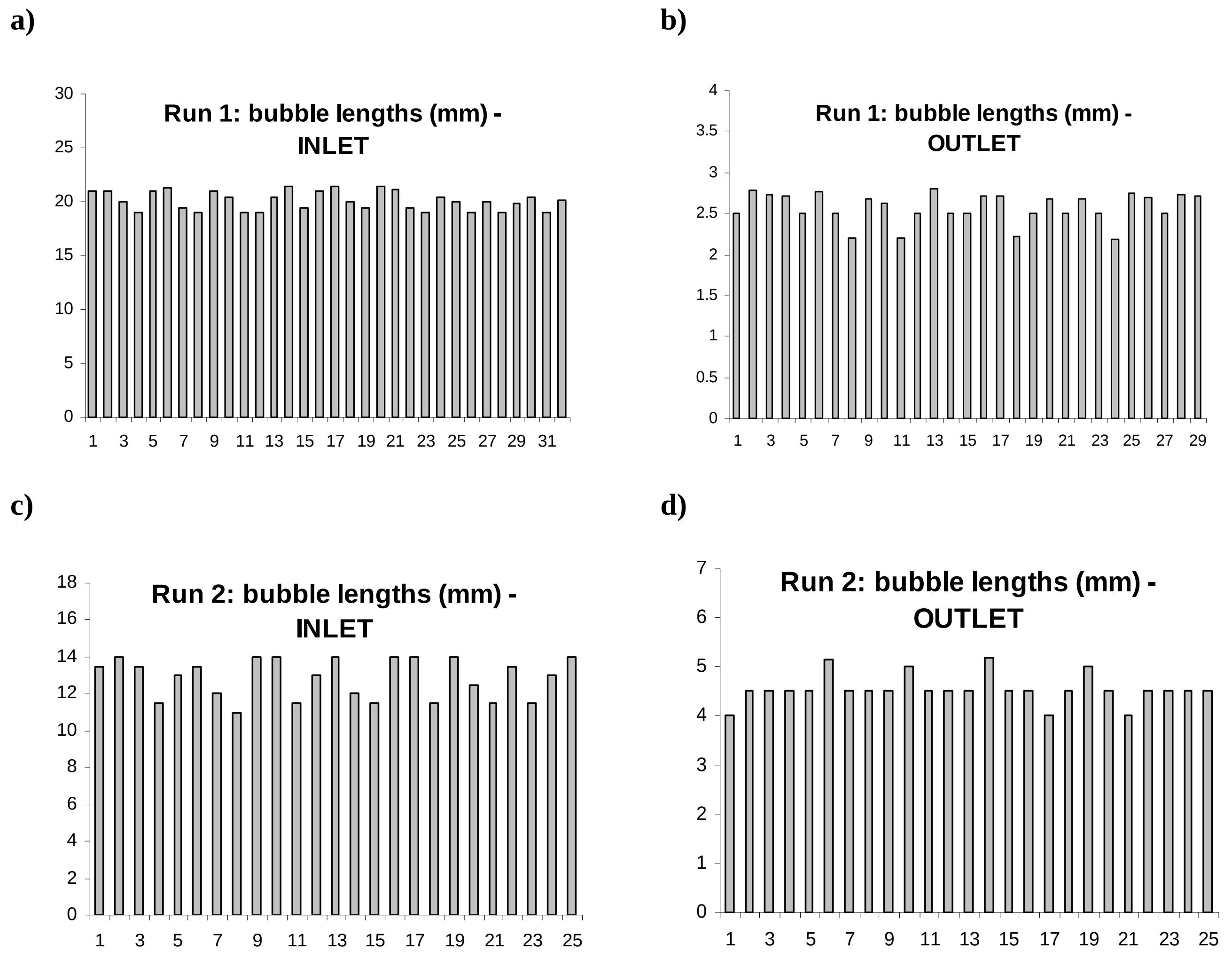

Figure 2. H_2 bubble lengths in mm *vs* the number of bubbles measured during the hydrogenation of 3-methyl-1-pentyn-3-ol in a capillary. a) Run 1 inlet; b) Run 1 outlet; c) Run 2 inlet; d) Run 2 outlet.

[1] M.T. Kreutzer, F. Kapteijn, J.A. Moulijn, C.R. Kleijn, J.J. Heiszwolf, *AIChE J.* **2005**, 9, 2428.

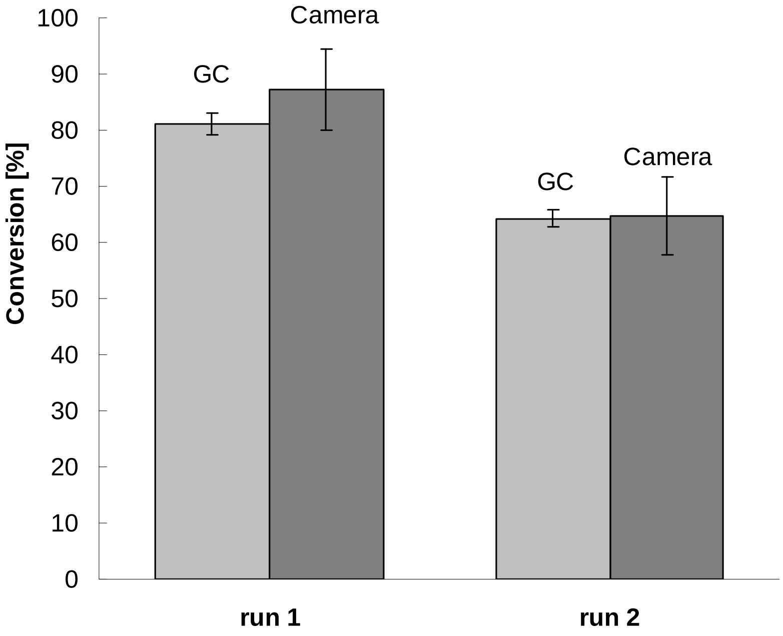

Figure 3. Comparison of 3-methyl-1-pentyn-3-ol conversion in a capillary, based on visual analysis of the H_2 bubble length evolution and the conversion determined by GC-analysis.

4. Cyclohexene hydrogenation: determination of kinetic parameters

Cyclohexene hydrogenation experiments were analyzed using Varian CP-3380 GC with a Chrompack CP-SIL 8 CB (PBX5) column of 60 m (internal diameter 0.25 mm; injector temperature = 200°C; ramp = 10 K min^{-1}; FID temperature = 330°C). Retention times were: 4.5 min (cyclohexene), 4.3 min (cyclohexane), 9.2 min (n-decane), and 4.0 min (ethanol).

Cyclohexene (Aldrich, used as received without purification) hydrogenation was performed in a 0.2 m capillary with a Pd loading of 2.7 wt%. The cyclohexene feed concentration was varied from 30 to 600 mol m^{-3} in dry ethanol (Aldrich) and *n*-decane (Merck), which allowed us to determine the first-order part and the zero-order part of the Langmuir-Hinshelwood kinetic expression (Figure 4). The adsorption constant of cyclohexene was found to be $K \approx 0.01$ m^3 mol^{-1}. The kinetic parameters were obtained at low cyclohexene conversion (<20%), at intermediate conversion levels (20% to 70%) or at high conversion levels (>70%). In all cases, the same values were obtained for the kinetic constants, indicating that axial dispersion or back-mixing had no negative impact on the measurement of kinetics.

Reactions were performed with temperatures up to 90°C. It was found that the observed activation energy decreased at temperatures above 55°C to a new value of 14 kJ mol^{-1}, indicating that at higher temperatures diffusion inside the γ-Al_2O_3 layer was starting to limit the reaction rate.

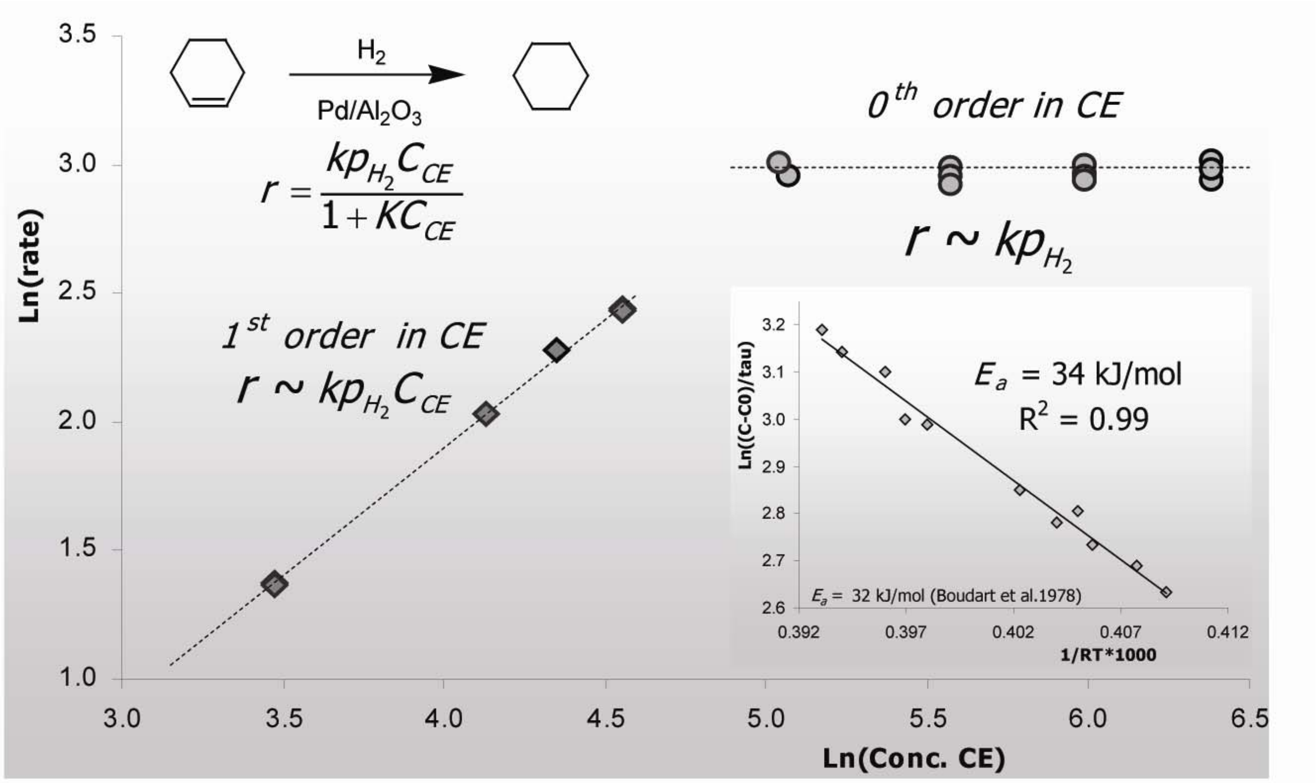

Figure 4. Determination of the kinetic parameters - rate constant *k*, adsorption constant *K*, and activation energy E_A: logarithmic cyclohexene hydrogenation rate *vs* logarithmic initial cyclohexene concentration in a capillary (2.7 wt% Pd).

5. 3-phenyl-propyl-azide hydrogenation: determination of catalyst activity, selectivity, and lifetime for differently prepared reaction mixtures

Conversion of 3-phenyl-propyl-azide **4** (Scheme 2, Communication) into 3-phenyl-propyl-amine **5** was detected with GC (a HP6890 with a CP-SIL 5CB-MS column) and HPLC (HP1050 with a Machery-Nagel ET 250/4 Nucleosil 120-5-C18 column). From samples taken prior to hydrogenation the following compounds could be identified by HPLC: the solvent (dry ethanol, toluene, or NMP (Aldrich)), 3-phenyl-propyl-azide **4** (14.8 min), 3-phenyl-propanol **1** (3.8 min), 3-phenyl-propyl-mesylate **3** (5.8 min), 3-

phenyl-propyl-chloride **2** (16.0 min) and NaN_3 (2.6 min). After hydrogenation, the HPLC chromatograms showed several new peaks. The main new peak (2.1 min) was identified as the primary amine 3-phenyl-propyl-amine **5**, as was verified by purchasing compound **5** (Aldrich) and running it through the HPLC.

After several hydrogenation runs at least two new peaks (retention times: 4.1 min and 4.9 min) appeared in the HPLC diagrams. The molar weight of a peak with a retention time of 4.9 min (determined by LC-DAD-MS) was 253 g mol^{-1} corresponding with the secondary amine di-(3-phenyl-propyl)-amine **5b**, which was formed at high reactant concentrations, high temperature, and longer time on stream. In samples taken from hydrogenation runs in ethanol a third peak was detected, which has a molecular weight of 163 g mol^{-1}. It was identified by using LC-DAD-MS as a secondary amine **5c** formed from condensation of **5** with the solvent ethanol. A fourth by-product (retention time = 4.1 min) was detected with a very small peak in the HPLC chromatograms. Most likely, this is a tertiary amine, as it was also found in batch experiments with long reaction times (20 h). Moreover, the conversion of **4** was followed by GC analysis. This analysis was performed by using a HP6890 with a CP-SIL 5CB-MS column.

4 was synthesised by azidation of **3**, derived from **1**, or by azidation of 3-phenyl-propyl-chloride **2**. **3** was synthesized by reacting **1** with methane sulphonyl-chloride (Fluka) in CH_2Cl_2 (Aldrich). Aqueous NaN_3 (Janssen Chimica) reacted with **3** in NMP. NMP is a very polar solvent that dissolves NaN_3 and that provides some homogeneity. The azidation was performed in a batch reactor at 55°C and in a continuous-flow stainless-steel tubular microreactor at 110°C, and both gave >95% yield of **4** (Table 2, used as reactant in entry 1-2).

Alternatively, **3** was dissolved in acetone and contacted with aqueous NaN_3 at 52°C. The acetone was removed and extra water and diethyl ether were added. Subsequently, a solvent switch to ethanol resulted in a 1.04 wt% solution of **4** in ethanol with as impurities NaN_3, **3** (0.04 wt%), **1** (0.03 wt%), diethylether (0.8 wt%), water (0.09 wt%), and acetone (0.13 wt%) (Table 2, entry 2-5).

A solution of **4** in toluene was prepared by contacting **3** in toluene and a mixture of ammonium chloride in propanol/water with aqueous NaN_3 at 82°C. Subsequently, adding toluene resulted in phase separation, the toluene was washed with water and azeotropic distillation resulted in a solution of **4** in

toluene (Table 2, entry 6). **2** (Aldrich) was used without further purifications and dissolved in ethanol. Azidation was performed by reacting **2** with aqueous NaN_3 in a continuous-flow steel microreactor at temperatures up to 200°C to 100% yield of **4**. The liquid flow rate was varied from 100 to 300 μl min^{-1}. The H_2 flow rate was varied between 0 and 830 μl(STP) min^{-1}.

Table 2. Summary of the hydrogenation of 3-phenyl-propyl-azide **4** in Pd-capillaries operated in segmented flow over a 5.7 wt% Pd catalyst at 24°C and 1.1 bar H_2.

Entry	Reaction mixture<br>Solvent<br>Conc. /mmol l^{-1}	Gas and liquid flowrate /ml min^{-1}	Capillary length /cm<br>Residence time /s	Selectivity to **5** /%<br>at<br>conversion of **4** /%	Pd half-life /min<br>TON[a]
1	NMP[d]<br>57 **4**; 1.0 **3**; 3 NaN_3	0.3<br>0.2	45<br>11.4	100<br>60	15[g]<br>196
2	NMP[d]<br>76 **4**; 5.3 **3**; 16 NaN_3	0.6<br>0.2	43<br>6.9	100<br>53	6[g]<br>92
3	EtOH[e]<br>52 **4**; 1.5 **3**; 4 NaN_3	0.6<br>0.25	49<br>7.4	100<br>96	60<br>1815
4[b]	EtOH[e]<br>52 **4**; 1.5 **3**; 4 NaN_3	0.8<br>0.25	49<br>5.9	86<br>100	140<br>2911
5	EtOH[e]<br>94 **4**; 3.0 **3**; 7 NaN_3	0.6<br>0.25	49<br>7.4	49<br>89	33<br>1089
6	Toluene[e]<br>54 **4**; 4.9 **3**; 4 NaN_3	0.6<br>0.25	49<br>7.4	96<br>87	15<br>298
7	EtOH/H_2O[f]<br>56 **4**; 11 **2**; 139 NaN_3	0.6<br>0.1	29<br>5.6	82<br>70	>205<br>>>2000[c]
8	EtOH/H_2O[f]<br>154 **4**; 5 **2**; 55 NaN_3	0.8<br>0.15	21<br>2.7	95<br>32	123<br>3816
9	EtOH/H_2O[f]<br>154 **4**; 5 **2**; 55 NaN_3	0.4<br>0.3	14<br>2.6	87<br>13	70<br>2697

[a] time, turnover number when conversion of **4** has dropped to 50% of initial value; [b] 61°C; [c] only 7% drop in selectivity after 205 min; [d] > 10% H_2O; [e] < 5% H_2O; [f] EtOH:H_2O=6:4; [g] Pd leaching occurred.

Summarizing, two different 3-phenyl-propyl-azide synthesis routes were applied: via mesylate and chloride intermediates. Several solvents or mixtures of solvents were used. There was variation of impurities in the reaction mixtures prior to hydrogenation that can influence reaction rates and stability. The concentrations of impurities were different for different prepared batches. Most hydrogenation runs were performed at 24°C and a H_2 pressure of 1.1 bar. Several runs were conducted at 61°C (Table 2, entry 4). Figure 5 shows two typical hydrogenation results in a capillary of the entries 3 and 8 (Table 2).

For the mesylate-derived 3-phenyl-propyl-azide **4**, >99% selectivity was observed for all WHSV*TOS. However, the catalyst activity decreased to 2% after a WHSV*TOS of 2. The chloride-derived 3-phenyl-propyl-azide **4** shows an increased catalyst lifetime and still retained 12% activity after WHSV*TOS of 9. However, a drop in selectivity to 3-phenyl-propyl-amine **5** is from 95 to75% with increasing TOS. The activity could be increased to 30% (without any further loss of activity with TOS) after regeneration of the Pd catalyst in flowing N_2(g) at 200°C for 2h (not shown).

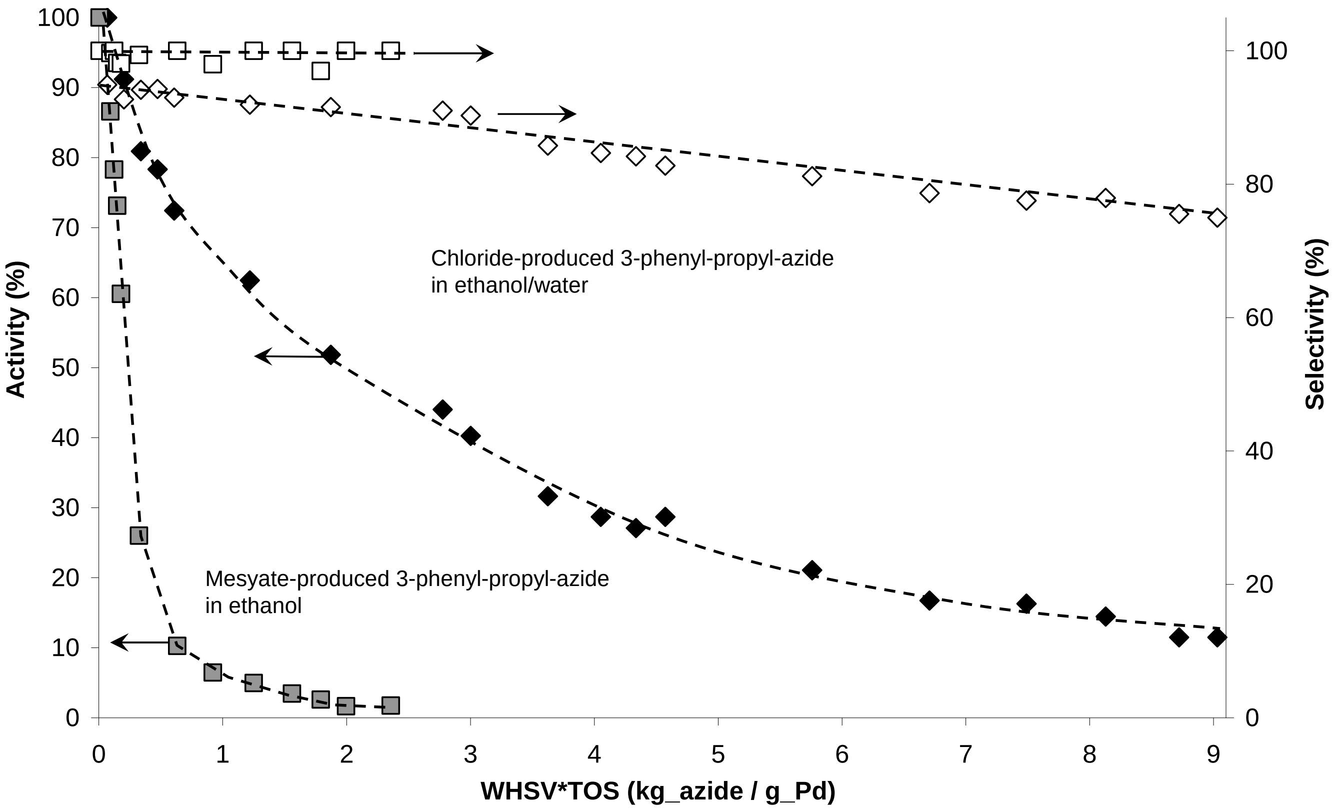

Figure 5. Normalized activity (% initial conversion) in 3-phenyl-propyl-azide hydrogenation and selectivity towards 3-phenyl-propyl-amine **5** *vs WHSV*TOS* (kg_{azide} / g_{Pd}) in a capillary (5.7 wt% Pd). Key: black lozenges: 2.85 wt% chloride-produced 3-phenyl-propyl-azide in ethanol/water: u_G/u_L = 5.5, u_L = 150 µl min^{-1}, L_{CAP} = 0.21 m, T = 24°C, X_{azide} = 32%; Open lozenges: selectivity to 3-phenyl-propyl-amine **5**; Grey squares: 1.04 wt% mesylate-produced 3-phenyl-propyl-azide in ethanol: u_G/u_L = 2.4, u_L = 250 µl min^{-1}, L_{CAP} = 0.49 m, T = 24°C, X_{azide} = 96%. Open squares: selectivity to 3-phenyl-propyl-amine **5**. The squares correspond to entry 3 and the lozenges to entry 8 in Table 2. The dashed lines are guidance for the eyes.
